# Automatic Modulation Classification Based on CNN-Transformer Graph Neural Network

**DOI:** 10.3390/s23167281

**Published:** 2023-08-20

**Authors:** Dong Wang, Meiyan Lin, Xiaoxu Zhang, Yonghui Huang, Yan Zhu

**Affiliations:** 1 Key Laboratory of Electronics and Information Technology for Space Systems, National Space Science Center, Chinese Academy of Sciences, Beijing 100190, China; wangdong20@mails.ucas.ac.cn (D.W.); linmeiyan18@mails.ucas.ac.cn (M.L.); zhangxiaoxu19@mails.ucas.ac.cn (X.Z.); yonghui@nssc.ac.cn (Y.H.); 2 University of Chinese Academy of Sciences, Beijing 100049, China

**Keywords:** deep learning, modulation classification, graph neural network, transformer network

## Abstract

In recent years, neural network algorithms have demonstrated tremendous potential for modulation classification. Deep learning methods typically take raw signals or convert signals into time–frequency images as inputs to convolutional neural networks (CNNs) or recurrent neural networks (RNNs). However, with the advancement of graph neural networks (GNNs), a new approach has been introduced involving transforming time series data into graph structures. In this study, we propose a CNN-transformer graph neural network (CTGNet) for modulation classification, to uncover complex representations in signal data. First, we apply sliding window processing to the original signals, obtaining signal subsequences and reorganizing them into a signal subsequence matrix. Subsequently, we employ CTGNet, which adaptively maps the preprocessed signal matrices into graph structures, and utilize a graph neural network based on GraphSAGE and DMoNPool for classification. Extensive experiments demonstrated that our method outperformed advanced deep learning techniques, achieving the highest recognition accuracy. This underscores CTGNet’s significant advantage in capturing key features in signal data and providing an effective solution for modulation classification tasks.

## 1. Introduction

Wireless communication has become indispensable in various national undertakings and the daily routines of individuals. In light of the rapid advancements in wireless communication technology, achieving efficient data transmission in complex wireless environments has become imperative. Various modulation schemes are commonly employed to modulate transmitted signals, and the diversity and intricacy of modulation methods continue to expand. As an intermediate step between signal reception and demodulation, automatic modulation classification (AMC) is a critical technology in military and civilian communications [1,2]. It finds application in electronic warfare, intelligence operations, surveillance, threat analysis, and spectrum monitoring, facilitating the identification of modulation schemes and the subsequent decoding of received signals. Therefore, a reliable and effective AMC scheme is of paramount importance.

After extensive research and development, researchers categorized AMC methods into three main groups: likelihood-based (LB) methods, distribution-test-based (DT) methods, and feature-based (FB) methods. The LB [3,4,5,6] methods involve comparing observed signal samples with various modulation hypotheses, to estimate their similarity, leading to the derivation of a likelihood function based on the selected signal model. Classification decisions are then made by assessing the resemblance to different modulation hypotheses. However, this method often necessitates prior knowledge of channel parameters and other assumptions, resulting in increased computational complexity of the likelihood function.

The DT methods adhere to the symbol mapping mechanism framework [7], tailored for each specific supported modulation scheme. When the theoretical distributions of diverse modulation patterns are accessible, the goal is to identify the distribution that best aligns with the signal to be classified. The classification procedure involves determining the optimal goodness of fit (GoF) [8] among the hypothesized signal distributions. However, this method exhibits subpar recognition performance under low signal-to-noise ratio (SNR) conditions.

FB methods are carried out in two sequential steps: feature extraction and classifier design. These methods commonly employ various types of features, such as instantaneous features (e.g., instantaneous frequency, phase, and magnitude [9]), statistical characteristics (e.g., higher-order cumulants and higher-order moments [10]), and wavelet transform features [11]. Essentially, the FB approach segregates signals of different modulation modes within a high-dimensional feature space by designing an appropriate classifier. The choice of classifiers includes support vector machines (SVM) [12], decision trees [13], hidden Markov models [14], and artificial neural networks [15]. However, noise is often present in practical, complex, and dynamic communication environments, and manual feature extraction may need help to select the most discriminative features. Moreover, the classifiers designed are often not optimally tailored to the selected artificial features. These challenges can result in a diminished classification performance when facing real-world conditions.

The aforementioned LB and DT methods necessitate prior knowledge of specific channels and noises, and practical application scenarios often involve numerous uncertain factors, leading to a relatively low modulation recognition accuracy. Machine learning-based approaches extract meaningful attributes from raw data to describe and represent the data. These methods have shown remarkable performance in computer vision [16], natural language processing [17], recommendation systems [18], object detection [19], anomaly detection [20,21,22], and other domains. In recent years, the field of deep learning has witnessed remarkable progress, giving rise to a diverse range of network models, such as convolutional neural network (CNN) [23], recurrent neural network (RNN) [24], graph neural network (GNN) [25], and transformer network [26] models. These models have demonstrated exceptional achievements in the aforementioned applications. Consequently, modulation recognition methods based on deep learning have garnered increasing attention. In contrast to manual feature extraction, deep-learning-based methods seamlessly integrate feature extraction with classifier design, enabling automatic feature extraction and classification through an end-to-end model. For example, O’Shea et al. [27] utilized a CNN architecture comprising two convolutional layers and two fully-connected layers to extract distinctive features from raw IQ signals. Moreover, they extended their work in [28] by incorporating a 1D CNN to enhance the residual network’s performance compared to the VGG network. Remarkably, when SNR exceeds 5 dB, this approach demonstrates a favorable classification effect. Another notable approach, proposed by Hong et al. [29], introduced a modulation classification method based on RNNs utilizing gated recurrent units (GRUs). This method surpassed the recognition performance of certain CNN models. Furthermore, Sreeraj et al. [8] achieved high recognition accuracy by converting the IQ signal into magnitude and phase representations, and feeding them into an LSTM (long short-term memory) network.

Due to the successful application of GNNs, there has been a growing interest in converting time series into graph structures and utilizing GNNs for classification. Transforming signal sequences into graph structures allows modeling direct relationships between sample points. Compared to directly extracting raw IQ signal features using the aforementioned deep learning methods, this approach can extract potential discriminative features that might be challenging to obtain from the original IQ signals. Furthermore, the transformer network [26], initially introduced in natural language processing, has demonstrated significant advantages in handling sequential data. Therefore, by combining the transformer’s ability to capture dynamic relationships between long sequences and the remarkable benefits of GNNs in processing graph-structured data, in this paper, we propose a novel learnable architecture for modulation classification. This architecture combines a CNN, transformer, and GNN, enabling flexible construction of graph structures between different sample points. First, we introduce the CNN-transformer network (CTN), which adaptively maps raw IQ signals into graph structures, rather than using fixed mapping rules. Fixed mapping rules make constructing an optimal graph structure for signals challenging. In contrast, our proposed mapping method provides greater flexibility, allowing the learned graph structure to be adjusted for optimization, resulting in an improved modulation classification performance. This mapping reveals inherent complex relationships between different signal segments and models their topological connections, enabling a more accurate understanding of the intrinsic features of modulation schemes and enhancing the model’s ability to recognize unlabeled modulation types. Second, we construct a GNN architecture based on GraphSAGE [30] and DMoNPool [31]. This architecture leverages the graph structure obtained from CTN to perform the classification task. The CTN and GNN form our modulation classification framework, called a CNN-transformer graph neural network (CTGNet). The main contributions of this paper are as follows:

- We preprocess the IQ signal using a sliding window and reorganize the subsequences into a signal matrix; we propose the CTN, which can dynamically map the subsequence matrix into a graph structure, thereby mining temporal and spatial relationships of signal data, to better understand modulation schemes;
- We integrate CTN and GNN for end-to-end training and optimization, which can automatically learn the most reasonable graph representation of signals, and the network structure can be flexibly extended for application to different classification tasks;
- We validated our method using the publicly available datasets RML2016.10a and RML2016.10b. Comparisons with CNN, RNN, and other GNN-based models demonstrated that our CTGNet achieved state-of-the-art performance.

## 2. Related Works

### 2.1. Transformer in Classification

The transformer network, introduced by Vaswani et al. [26], is a neural network architecture based on the attention mechanism, initially designed for sequence modeling in natural language processing tasks. Unlike traditional RNNs and CNNs, the transformer’s attention mechanism can simultaneously consider information from different positions in the sequence, making it highly effective for handling long-range dependencies and capturing global context. To extend the applicability of the transformer to image classification tasks, the vision transformer (ViT) [32] approach was proposed. ViT divides the image into a set of fixed-size image patches and treats each patch as a sequence input to the transformer. This enables ViT to capture global relationships within images, free from the constraints of receptive fields. Building on this, the transformer-in-transformer (TNT) [33] method incorporates a small transformer module within each image patch to capture local features. This enables interaction between local features and fine-grained relationship modeling across the entire image. Another advancement is the swin transformer [34], which adopts a hierarchical transformer architecture and performs self-attention modeling within each image patch. By interacting with image patches at different levels, a swin transformer can effectively capture multi-scale features in images, enhancing its modeling capabilities. Integrating various attention mechanisms, hierarchical structures, and feature interaction approaches in these models extends and improves the transformer architecture, leading to remarkable performance in image classification tasks. The successful application of these transformer-based models showcases their broad adaptability and powerful representation learning capabilities.

### 2.2. GNN in Classification

GNNs are a specialized category of neural network models specifically designed to handle graph-structured data. GNNs exhibit exceptional capabilities in learning and inferring from nodes and edges within a graph, enabling them to effectively capture intricate relationships and local patterns. A prominent example of a GNN are the graph convolutional networks (GCNs) proposed by Kipf et al. [35], which adapt convolutional neural network concepts to graph data. By iteratively aggregating information from neighboring nodes, GCNs update node representations, facilitating information propagation and feature extraction on graphs. In the quest for scalability, Hamilton et al. [30] introduced GraphSAGE, incorporating sampling and aggregation strategies to handle large-scale graph data, thus enhancing the model’s scalability without compromising its effectiveness. Another noteworthy advancement is the graph attention networks (GAT) introduced by Velickovic et al. [36]. GAT leverages attention mechanisms to learn node weights, allowing for more accurate capturing of node relationships and enhancing the model’s expressive power. To address the issue of graph dimensionality, Ying et al. [37] proposed DIFFPOOL, a differentiable pooling method that employs a hierarchical clustering strategy to partition nodes into subgraphs. This strategy effectively reduces the dimensionality of the entire graph, while preserving crucial structural information. Furthermore, deep modularity networks (DMoN), proposed by Anton et al. [31], are graph pooling methods that utilize a modularity measure. DMoNPool selects pooling nodes by optimizing modularity scores, enabling efficient learning and inference on graph data by capturing complex relationships effectively. Overall, these models demonstrate remarkable capabilities in handling graph-structured data through their effective relationship capture, incorporation of attention mechanisms, and employing pooling strategies for dimensionality reduction.

### 2.3. Deep-Learning-Based AMC

In recent years, deep learning methods have been extensively applied in the field of modulation classification, resulting in significant advancements. These methods effectively integrate feature engineering with classifier training, improving performance. Zhang et al. [38] first estimated the phase offset of the raw input signal, transformed the original signal based on this estimation, and then utilized the transformed signal for modulation classification. Inspired by hybrid models’ powerful feature extraction capabilities and the complementary information present in separate IQ channels, Xu et al. [39] proposed a novel multi-channel deep learning model named MCLDNN. This model extracts features from individual and combined IQ signals of the received data, considering the spatial and temporal aspects, and enhancing the classification performance. Yashashvi et al. [40] introduced a method to correct distorted signals by removing random frequency and phase offsets and then applied a CNN for feature extraction on the corrected signal. Liu et al. [41] designed a seven-layer residual network [42] for identifying ten types of modulation, showcasing the potential of deep learning in modulation classification. Tan et al. [43] employed a GRU to exploit temporal information for signal identification in exploring the temporal correlation feature. However, the direct use of a GRU network demonstrated comparable performance to that of a basic CNN in the work by O’Shea et al. [27]. Hu et al. [44] introduced an attention mechanism-based classifier framework, incorporating an RNN model with an attention mechanism to enhance classification performance. Zhang et al. [45] constructed a dual-stream network structure based on CNN and RNN, effectively extracting spatial and temporal-related features from the received signal. Furthermore, Sreeraj et al. [8] achieved a high recognition accuracy by transforming IQ signals into amplitude and phase representations and inputting them into an LSTM network, showcasing the benefits of using LSTM in modulation classification tasks. In another study detailed in [46], a novel approach was introduced to combine I/Q and A/P information. This method introduced a unique step attention fusion network (SAFN) module that amalgamates the diverse step outputs generated by the bidirectional gated recurrent unit (BiGRU) layer, each with distinct weights. Ghasemzadeh et al. [47] proposed an automatic modulation classification architecture and designed a stacking quasi-recurrent neural network (SQRNN) to simulate recurrent layer operations, and aggregate and extract features in time steps, thereby achieving high classification accuracy. In [48], the authors introduced a learning framework using an LSTM denoising auto-encoder to automatically extract reliable features from noisy radio signals for identifying modulation type. Zhang et al. [49] used fully-connected layers to build an autoencoder to enhance the information interaction between in-phase and quadrature channels, and the model learned the extracted interaction features and IQ data together. In [50], a hybrid model combining RNN and CNN, namely recurrent and convolutional neural network (R&CNN) was proposed. The model takes advantage of recurrent layers for time series data, as well as the spatial learning ability of convolutional layers. To solve the vulnerability of time–frequency representation to noise, Xu et al. [51] proposed a novel approach called deep TFT classification network (DTFTCNet), which integrates a time–frequency transform (TFT) and CNN within an end-to-end deep learning framework for radar signal modulation recognition. A modulation classification method based on deep feature fusion was proposed in [52], which utilizes ResNeXt for semantic feature extraction, GRU for time series representation, and combines ResNeXt with GRU output using discriminant correlation analysis (DCA) feature fusion models to improve AMC performance. Che et al. [53] introduced a spatial-temporal hybrid feature extraction network (STHFEN), which employs a dual feature extraction network to transform the signal into the spatial and temporal feature space and utilizes a hybrid inference classifier to combine the classification results. Zhang et al. [54] proposed high-order convolutional attention networks (HoCANs), which leverage a novel high-order attention mechanism to enhance feature correlations in radio signals. A complex-valued depthwise separable convolutional neural network (CDSCNN) was proposed in [55] for modulation classification. CDSCNN uses complex-valued operation units to achieve complex-valued feature learning. Zheng et al. [56] introduced a multi-scale radio transformer (Ms-RaT) with dual-channel representation to fuse frequency, amplitude, and phase information. A residual-attention convolutional network (RanNet) was proposed in [57], which employs advanced processing blocks, attention connections, and skip connections to enhance the intrinsic features of combined waveform data. Chang et al. [58] introduced a hierarchical classification head based convolutional gated deep neural network (HCGDNN) for modulation classification, leveraging the complementary information from different layers’ outputs in the deep learning model.

In addition to directly extracting features from raw time series, i.e., IQ signals, various methods have explored transforming IQ signals into alternative representations, such as images or matrices, to extract more discriminative features. Wang and Oates [59] utilized Gramian angular fields (GAF) and Markov transition fields (MTF) to convert time series into matrices, effectively capturing the underlying dynamics of the signals. Meanwhile, Peng et al. [60] transformed the original IQ signals into constellation diagrams and proposed an enhanced method for constellation diagram classification, leveraging AlexNet [61] and GoogLeNet [23] models for effective classification. Wang et al. [62] employed two CNN models for modulation classification. The first model identified seven modulation styles using raw IQ signals as input, while the second model, which we call ConsCNN, processed the input QAM constellation diagrams to recognize 16QAM and 64QAM modulations, demonstrating the significance of different signal representations in the classification task. Lin et al. [63] proposed a joint learning framework for robust modulation recognition in the presence of noise. The framework integrates three modules, dual-channel spectrum fusion, signal enhancement (SE), and signal classification (SC) into a single architecture, employing a multistage attention mechanism to enhance recognition-related features. In [64], a CNN-based time–frequency attention mechanism was proposed, focusing on learning meaningful frequency and temporal information for modulation recognition. Chen et al. [65] introduced the signal-to-matrix (S2M) method, where the I and Q channels underwent sliding window processing and were transformed into matrices. These matrices were then concatenated and fed into a CNN for feature extraction, showcasing the potential of matrix representations in feature learning. Statistical features have also been explored in modulation recognition. Lee et al. [66] designed a fully connected network using statistical features (such as skewness, kurtosis, and other high-order moments) as inputs, providing valuable insights into the significance of statistical characteristics in the classification process. On the other hand, Huang et al. [67] combined DenseNet [68] and LSTM to extract useful features from the cyclic correlation entropy vector (CCV) of the signal, highlighting the ability of deep learning models to capture intricate relationships within signals. Graph-based approaches have also gained traction. Liu et al. [69] treated each signal sample as a node in a graph and employed a CNN to learn node embeddings and adjacency matrices. Subsequently, graph convolutional networks were utilized for classification, demonstrating the power of graph representations in modeling complex relationships within the signals. Xuan et al. [70] used a graph neural network for modulation recognition, constructing an adjacency matrix through one-dimensional convolutions with different scales, to extract information between different sample points. Compared with the traditional methods of mapping time series to graphs (e.g., VG [71], HVG [72], LPVG [73]), their adaptive visibility graph algorithm (AVG) offered an adaptive way to convert the signal into a graph structure. However, this approach has limitations, as it relies on the size of the one-dimensional convolution kernel, overlooks the importance of global information, and cannot effectively capture long-distance dependencies within signals.

## 3. The Proposed Method

In this section, we comprehensively introduce CTGNet, describe the preprocessing of IQ signals, and utilize the CTN to generate graph representations of these signals. Furthermore, we also elucidate the architecture of GNN, which was specially designed for the feature extraction and classification of graphs. Before delving into the intricacies of our approach, it is worth highlighting two key considerations behind our proposed method. First, RNNs face the problem of vanishing and exploding gradients when dealing with long-term sequences. Furthermore, CNNs, while effective in capturing local information, may ignore critical global dependencies in the data. To circumvent these limitations, we leverage transformer networks, known for their remarkable ability to model long-range dependencies. By adopting a multi-head self-attention mechanism, a transformer network can directly identify the dependency between any two positions in the sequence. This property has proven invaluable in capturing the inherently multilevel information and multimodal features of signal sequences. Transformer networks thus become a compelling choice for modeling the underlying connections between different signal sequences, and these relationships are effectively represented as interactions between nodes in a graph structure. Furthermore, by building GNNs, we can better understand the topological relationships within the signal graph structure and the complex interaction patterns between nodes. This enhanced understanding facilitates the analysis of different modulation schemes. Considering all these factors, our CTGNet overcomes the limitations of traditional network models and fully exploits the potential of rich temporal information in time series data.

### 3.1. IQ Data Preprocessing

**Figure 1:**
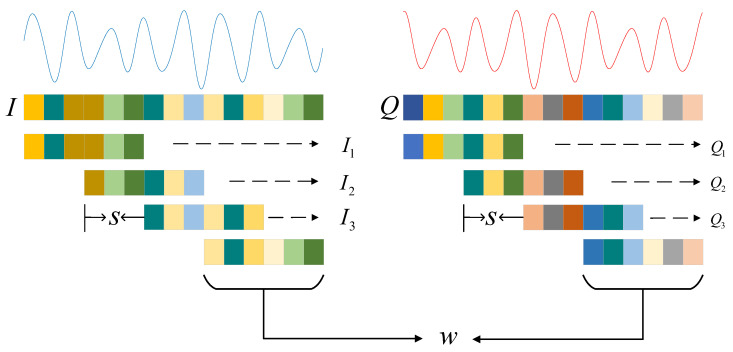
Data preprocessing flow of the *I* signal and *Q* signal. *w* is the length of the sliding window, and *s* is the step size of the sliding window.

The raw IQ data represents samples of the signals’ in-phase (I) and quadrature (Q) components. However, a transformer network requires an input in the form of embeddings. To achieve this, we employ sliding window processing to partition the long IQ sequence into shorter subsequences, each with a fixed length. Additionally, we specify the overlap length between adjacent subsequences. After partitioning the signal, we reorganize the subsequences into a signal sequence matrix and apply one-dimensional convolutional encoding. This encoding process transforms the subsequences into embeddings, capturing their essential features, which serve as input to the transformer network. The specific preprocessing steps are illustrated in Figure 1. A more detailed description of the steps is provided below.

The *I* signal and *Q* signal can be represented as $I=[i_{1},i_{2},\ldots ,i_{n}]$ and $Q=[q_{1},q_{2},\ldots ,q_{n}]$, where *I* and *Q* are one-dimensional vectors of length *n*. We partition both the *I* signal and *Q* signal into shorter subsequences of length *w*. The subsequences are generated with a sliding window of step size *s*:

(1) $$I_{1}=\left[i_{1},i_{2},\ldots ,i_{w}\right]$$

(2) $$I_{2}=\left[i_{1+s},i_{2+s},\ldots ,i_{w+s}\right]$$

…

(3) $$I_{m}=\left[i_{1+(m-1)\cdot s},i_{2+(m-1)\cdot s},\ldots ,i_{w+(m-1)\cdot s}\right]$$

(4) $$Q_{1}=\left[q_{1},q_{2},\ldots ,q_{w}\right]$$

(5) $$Q_{2}=\left[q_{1+s},q_{2+s},\ldots ,q_{w+s}\right]$$

…

(6) $$Q_{m}=\left[q_{1+(m-1)\cdot s},q_{2+(m-1)\cdot s},\ldots ,q_{w+(m-1)\cdot s}\right]$$

where $I_{1},I_{2},\cdots ,I_{m}$ are the subsequences partitioned by the *I* signal, and $Q_{1},Q_{2},\cdots ,Q_{m}$ are the subsequences partitioned by the *Q* signal. The number of overlapping elements between two adjacent subsequences is w−s. Therefore, for the *I* and *Q* signals of length *n*, the number of subsequences partitioned by the sliding window is *m*:

(7) $$m=\left\lfloor \frac{n-w}{s}+1\right\rfloor$$

After performing this partitioning operation, to maintain the time characteristics between adjacent subsequences and further explore their internal relations, the subsequences are rearranged to form an m×w matrix. For the *I* signal, the resulting matrix $X_{I}$ is represented as

(8) $$\begin{array}{c} X_{I}=\left[I_{1};I_{2};\ldots ;I_{m}\right]=\left[\begin{array}{cccc} i_{1} & i_{2} & \cdots & i_{w}\\ i_{1+s} & i_{2+s} & \cdots & i_{w+s}\\ \vdots & \vdots & \ddots & \vdots \\ i_{1+(m-1)\cdot s} & i_{2+(m-1)\cdot s} & \cdots & i_{w+(m-1)\cdot s} \end{array}\right] \end{array}$$

and for the *Q* signal, the resulting matrix $X_{Q}$ is represented as

(9) $$\begin{array}{c} X_{Q}=\left[Q_{1};Q_{2};\ldots ;Q_{m}\right]=\left[\begin{array}{cccc} q_{1} & q_{2} & \cdots & q_{w}\\ q_{1+s} & q_{2+s} & \cdots & q_{w+s}\\ \vdots & \vdots & \ddots & \vdots \\ q_{1+(m-1)\cdot s} & q_{2+(m-1)\cdot s} & \cdots & q_{w+(m-1)\cdot s} \end{array}\right] \end{array}$$

The previous steps described the preprocessing of the IQ data, including using sliding window operations to obtain subsequences and rearranging them into a subsequence matrix. In the following subsection, we introduce the process of mapping this matrix onto a graph structure.

### 3.2. Mapping the Processed Data to Graph

**Figure 2:**
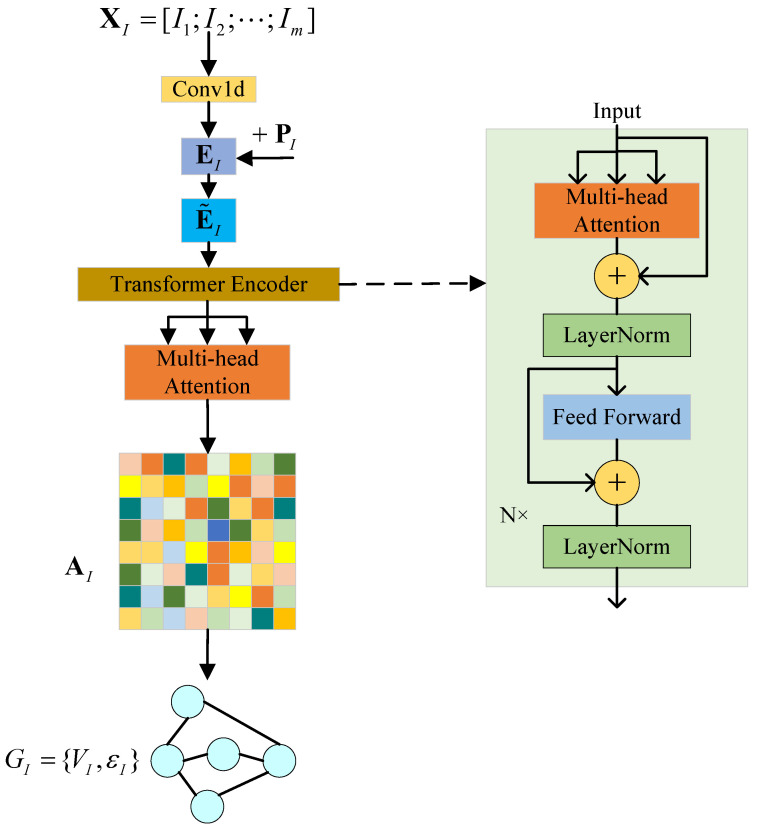
The architecture of CTN. This illustration demonstrates the architecture, using the I signal as an example. $G_{I}$ is the graph structure of the *I* signal obtained through CTN. Correspondingly, the graph structure of the *Q* signal is $G_{Q}$.

After obtaining the subsequence matrices $X_{I}$ and $X_{Q}$ for the IQ data, in this section, we propose the CTN used to map them onto corresponding graphs, namely $G_{I}=\left\{V_{I},\varepsilon_{I}\right\}$ and $G_{Q}=\left\{V_{Q},\varepsilon_{Q}\right\}$. The overall architecture of the CTN is illustrated in Figure 2.

Since the inputs of the transformer network are embeddings, after obtaining the matrices $X_{I}$ and $X_{Q}$, we encode them using one-dimensional convolution to obtain the corresponding embeddings $E_{I}$ and $E_{Q}$. Here, we assume that the dimension of the embeddings after one-dimensional convolution is *d*. For the input subsequence matrix of m×w, the output dimension after encoding is m×d. Next, in order to enable the model to capture the relative position and order information between subsequences and enhance the model’s ability to capture complex relationships between subsequences, we obtain ${\tilde{E}}_{I}$ and ${\tilde{E}}_{Q}$ by adding a learnable position encoding matrix $P_{I}$ and $P_{Q}$, both with dimensions of m×d,

(10) $${\tilde{E}}_{I}=E_{I}+P_{I}$$

(11) $${\tilde{E}}_{Q}=E_{Q}+P_{Q}$$

and in this way, ${\tilde{E}}_{I}$ and ${\tilde{E}}_{Q}$ will serve as the input to the transformer encoder.

**Figure 3:**
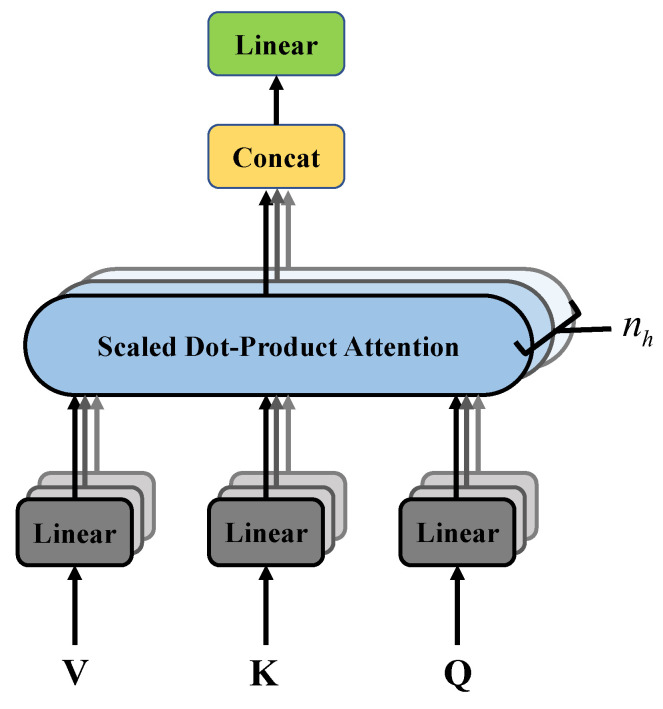
The architecture of the multi-head attention. V, K, and Q are the value matrix, key matrix, and query matrix obtained through linear transformation, respectively, and $n_{h}$ is the number of heads of multi-head attention.

The transformer encoder consists of multiple identical encoder layers containing two sub-layers: a multi-head self-attention network and feed-forward neural network. It also includes residual connections and layer normalization, which help address the vanishing gradient problem when applying deep models. The feed-forward neural network is a simple fully connected layer structure. The structure of the multi-head attention is shown in Figure 3. In the self-attention mechanism, each element in the input sequence interacts with other elements and computes attention weights between each element and the others. This allows the model to focus on the relationships between different positions in the input sequence. Multi-head attention divides the attention computation into multiple heads, where the attention is computed independently in each head. This enables different heads to focus on different aspects of the input sequence, providing more comprehensive information. Therefore, by learning the multi-head self-attention of the input sequence matrix, we obtain the adjacency matrix for the graph structure. According to [26], the specific computation process is as follows: The same computation process is applied to both the *I* signal and *Q* signal. Next, we will use the *I* signal as an example to explain this process.

To perform a linear transformation on the input subsequence embeddings ${\tilde{E}}_{I}$ and based on the number of attention heads $n_{h}$, we multiply ${\tilde{E}}_{I}$ by weight matrix $W_{i}^{Q}$, $W_{i}^{K}$, and $W_{i}^{V}$, respectively. This operation yields the query matrix $Q_{i}$, key matrix $K_{i}$, and value matrix $V_{i}$ for each attention head:

(12) $$Q_{i}={\tilde{E}}_{I}W_{i}^{Q}$$

(13) $$K_{i}={\tilde{E}}_{I}W_{i}^{K}$$

(14) $$V_{i}={\tilde{E}}_{I}W_{i}^{V}$$

where *i* ranges from 1 to $n_{h}$. $W_{i}^{Q}$, $W_{i}^{K}$, and $W_{i}^{V}$ are weight matrices of dimensions $d\times d_{k}$, $d\times d_{k}$, and $d\times d_{v}$, respectively.

Then, by calculating the scaled dot-product between the query matrix $Q_{i}$ and the key matrix $K_{i}$ of each head, the correlation between them can be obtained. Through softmax function, the attention ${\mathrm{att}}_{i}$ of each head can be obtained, and the attention can be weighted and summed over the value matrix $V_{i}$ to produce the output ${\mathrm{head}}_{i}$:

(15) $${\mathrm{att}}_{i}=\mathrm{softmax}\left(\frac{Q_{i}K_{i}^{T}}{\sqrt{d_{k}}}\right)$$

(16) $${\mathrm{head}}_{i}={\mathrm{att}}_{i}V_{i}=\mathrm{softmax}\left(\frac{Q_{i}K_{i}^{T}}{\sqrt{d_{k}}}\right)V_{i}$$

and scaling makes the range of attention more appropriate, helping to train a stable model, and softmax makes all attention sum to 1.

In this way, each ${\mathrm{head}}_{i}$ focuses on different representations of information, and different heads can focus on the dependencies between different locations. After that, concatenate each ${\mathrm{head}}_{i}$ and multiply them with the matrix $W^{O}$ of size $n_{h}d_{v}\times d$ to obtain the matrix MultiHead:

(17) $$\mathrm{MultiHead}=\left[{\mathrm{head}}_{1},{\mathrm{head}}_{2},\cdots ,{\mathrm{head}}_{n_{h}}\right]W^{O}$$

and this multi-head attention mechanism facilitates the model’s comprehension of the input data from diverse perspectives, thereby enhancing its capacity to capture a more extensive range of information.

Finally, after adding the MultiHead to the input ${\tilde{E}}_{I}$ and passing through LayerNorm, the result is sent to the feed-forward neural network, so that the output of the transformer encoder is obtained. Here, each element in ${\mathrm{att}}_{i}$ represents the relative importance and correlation between each signal subsequence and other subsequences in the input subsequence matrix. By stacking the encoder layers as described above, we take the average value of the multi-head attention ${\mathrm{att}}_{i}$ from the last encoder layer as the adjacency matrix A of the graph,

(18) $$A=\frac{\sum_{i=1}^{n_{h}}{\mathrm{att}}_{i}}{n_{h}}$$

In addition, the input to the graph neural network includes the adjacency matrix and the node feature representation. For the node *j* in graph $G_{I}$ and $G_{Q}$, we concatenate the corresponding subsequences as the feature representation of the node, denoted as

(19) $$F_{j}=\left[i_{1+(j-1)\cdot s},i_{2+(j-1)\cdot s},\cdots ,i_{w+(j-1)\cdot s},q_{1+(j-1)\cdot s},q_{2+(j-1)\cdot s},\cdots ,q_{w+(j-1)\cdot s}\right]$$

We obtained the graphs of the I signal and the Q signal through the above steps. In the next subsection, we will use GNN for classification.

### 3.3. Classification of Graphs Using GNN

After mapping the I signal and Q signal onto graphs using the proposed CTN, we constructed a graph neural network for classification. The architecture of the graph neural network consists of GraphSAGE layers [30] followed by DMoNPool layer [31]. GraphSAGE is responsible for learning node representations in graph data. It achieves this by employing neighborhood sampling and feature aggregation. During this process, a set of neighboring nodes is sampled for each node, and their features are aggregated to generate a new representation for the central node. This operation is repeated for multiple iterations to progressively refine the node representations, capturing local and higher-order neighborhood information. On the other hand, DMoNPool handles the pooling of learned node representations to generate graph-level representations. By combining GraphSAGE and DMoNPool in CTGNet, the model can simultaneously leverage local node features and global graph structure features. After performing multi-layer node feature extraction and graph pooling, we obtained the feature vectors $T_{I}$ and $T_{Q}$ for the *I* signal and *Q* signal, respectively:

(20) $$T_{I}=\left[T_{i1},T_{i2},\cdots \right]$$

(21) $$T_{Q}=\left[T_{q1},T_{q2},\cdots \right]$$

and then concatenated $T_{I}$ and $T_{Q}$ to form:

(22) $$T_{IQ}=\left[T_{i1},T_{i2},\cdots ,T_{q1},T_{q2},\cdots \right]$$

so the concatenated feature vector $T_{IQ}$ is then fed into a final fully connected layer for classification.

**Figure 4:**
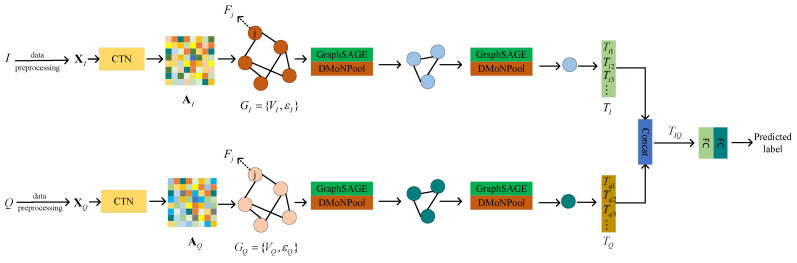
The architecture of the CTGNet. $A_{I}$ and $A_{Q}$ are the adjacency matrices of the graph structure of the *I* signal and *Q* signal, respectively. $F_{j}$ is the node feature. $T_{I}$ and $T_{Q}$ are the average feature vectors.

The above is our proposed CTGNet, as shown in Figure 4, and the information of each layer is shown in Table 1. It is able to learn graph-structured representations of signals in an end-to-end manner. By using CTN to map the *I* signal and *Q* signal to graphs $G_{I}$ and $G_{Q}$, we construct a graph neural network based on a GraphSAGE layer and DMoNPool layer. CTGNet employs a holistic training approach for integrating graph construction and classification processes, ensuring that the transformed graph structure is optimal for modulation classification. Furthermore, representing signals as graphs can learn topological relations and extract global information among different sample points.

## 4. Dataset and Experiments

### 4.1. Dataset and Parameter Setting

In this paper, we evaluated our proposed method using the RML2016.10a [27] and RML2016.10b [74] datasets. The RML2016.10a dataset considers the effects of real-world electromagnetic environments and consists of 11 common modulation schemes. Each modulation scheme generated 20,000 samples at 20 different SNR levels, resulting in a total of 220,000 samples. The RML2016.10b dataset includes 10 modulation schemes and comprises a total of 1.2 million samples. Each sample in both datasets is organized into 2 × 128. The specific parameters of the datasets are presented in Table 2.

For the experiments, we split the RML2016.10a dataset into 80% for training and 20% for testing. As for the RML2016.10b dataset, we used 60% of the data for training and 40% for testing. During the model training process, we employed the cross-entropy loss function and utilized an Adam optimizer with a learning rate of 0.001. The batch size was set to 128. All our experiments were executed on a Nvidia GeForce GTX 1080 GPU, and all models were implemented using the PyTorch deep learning framework.

### 4.2. Baseline Methods

We compared our proposed method with nine different deep network models in terms of modulation classification performance. These models include AvgNet [70], MCLDNN [39], Resnet1d, VGG [28], CNN2d [27], GRU [29], LSTM [8], GAF [59], and ConsCNN [62]. Among them, GRU and LSTM are based on RNN, while Resnet1d, CNN2d and VGG are based on CNN. MCLDNN combines RNN and CNN architectures. Regarding AvgNet, it adopts one-dimensional convolution of different scales to construct the adjacency matrix of the signal, thus realizing classification based on GNN. GAF converts the signal into images and uses a resnet network to extract image features. ConsCNN processes the constellation diagram of the signal to realize the identification of modulation types.

### 4.3. Evaluation Metrics

In this paper, we used accuracy, F1 score, and recall to measure the performance of the different models. The accuracy was formalized as the proportion of correct predictions for the entire test set, as shown in Equation (23):

(23) $$accuracy=\frac{\sum_{i=1}^{C}\left(TP_{i}+TN_{i}\right)}{\sum_{i=1}^{C}\left(TP_{i}+TN_{i}+FP_{i}+FN_{i}\right)}$$

where i=1,⋯,C.*C* is the number of sample categories included in the dataset and the notations of $TP_{i}$, $FP_{i}$, $FN_{i}$, $TN_{i}$ are described as the number of testing samples from *i*-th class in the conditions as below:

- True Positive (TP): Truly positive, predicted to be positive
- False Positive (FP): Truly negative, predicted to be positive
- False Negative (FN): Truly positive, predicted to be negative
- True Negative (TN): Truly negative, predicted to be negative

F1 Score is an indicator used to measure model performance in classification problems, defined as the harmonic mean of precision and recall. Therefore, the precision and recall of the i-th class prediction are shown in Equations (24) and (25):

(24) $$precison_{i}=\frac{TP_{i}}{TP_{i}+FP_{i}}$$

(25) $$recall_{i}=\frac{TP_{i}}{TP_{i}+FN_{i}}$$

and the total F1 score can be calculated as the mean of the F1 score in each class in Equation (26):

(26) $$F1=\frac{1}{C}\left(\sum_{i=1}^{C}\frac{2\times precision_{i}\times recall_{i}}{precision_{i}+recall_{i}}\right)$$

### 4.4. Results and Discussion

#### 4.4.1. Ablation Study

In this section, an ablation study was performed to observe the effect of data preprocessing on the performance of CTGNet. The recognition performance of the model is presented in Table 3 for two scenarios: without data preprocessing and with data preprocessing. The results indicated that the model equipped with data preprocessing achieved a higher recognition accuracy than the model without preprocessing and exhibited a reduced training time. Through sliding window preprocessing, the original lengthy signal sequence is partitioned into shorter subsequences, which are organized into a subsequence matrix. The advantage of this approach lies in CTGNet’s ability to learn correlations among distinct subsequences, while considering the extraction of intrinsic features within each subsequence. This effectively captures both local and global features. Thus, data preprocessing not only enhances the model’s capability to understand intricate inter-subsequence relationships, but also facilitates the extraction of rich intra-subsequence features. Simultaneously, the reduced number of nodes in the learned graph significantly streamlines the overall processing time. The experimentation above confirmed the superiority of our proposed data preprocessing method.

#### 4.4.2. Experiments on Different Sliding Window Sizes and Step Sizes

**Figure 5:**
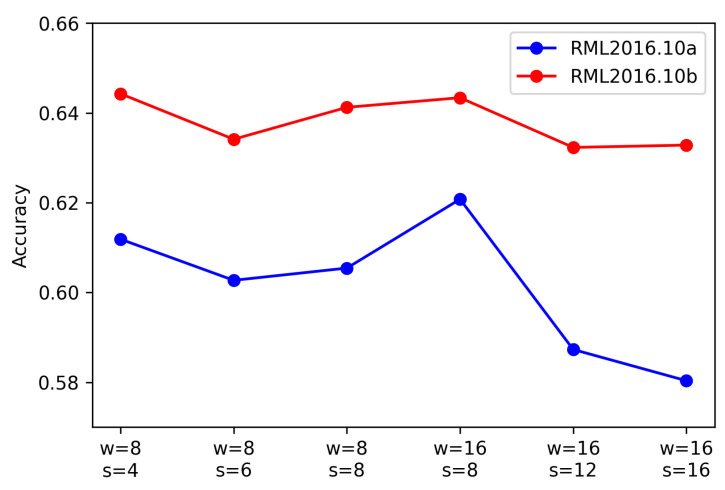
Recognition accuracy for the two datasets with different sliding window sizes and step sizes. Here, the sliding window sizes were 8 and 16, and the corresponding step sizes were 4, 6, and 8, and 8, 12, and 16, respectively.

In this section, different sizes of sliding windows and strides were used in the data preprocessing stage, resulting in various sizes of samples and different degrees of overlap between adjacent sequences. Considering that each sample in both datasets contained 128 data points, we used sliding windows of size 8 and 16, with strides of 4, 6, and 8, and 8, 12, and 16, respectively. Employing these different window sizes and strides has an impact on the subsequent mapping to graph structures and classification using graph neural networks. Figure 5 displays the experimental results on both datasets under different settings.

From Figure 5, it can be observed that a higher recognition accuracy was achieved on the 2016.10a and 2016.10b datasets when using a window size of 16 with a stride of 8, as well as with a window size of 8 and a stride of 4. This indicates that under these parameter combinations, the model was able to construct graph structures more effectively and capture discriminative features for classification.

#### 4.4.3. Comparisons with Other Baseline Methods

**Figure 6:**
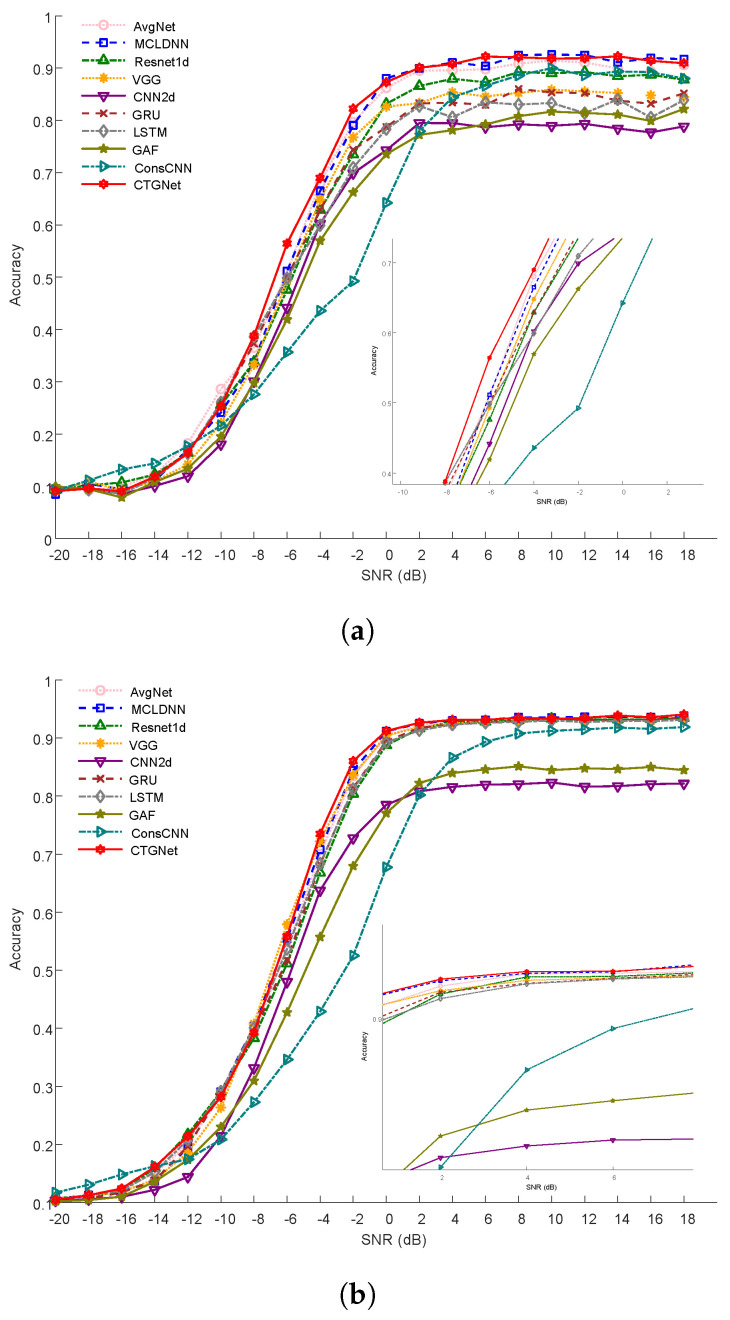
Recognition accuracy of ten methods with a change in SNR, where SNR ranged from −20 dB to 18 dB. (**a**) RML2016.10a; (**b**) RML2016.10b.

We compared our proposed model, which utilized a window size of 16 and a stride of 8, with various baseline methods. The experimental results of the two datasets are shown in Table 4 and Table 5, showing the accuracy, F1 score, and recall. In addition, Figure 6 shows the recognition accuracy of different methods as the signal-to-noise ratio changes. It is evident from the experimental results that our model achieved the highest performance on both datasets.

**Figure 7:**
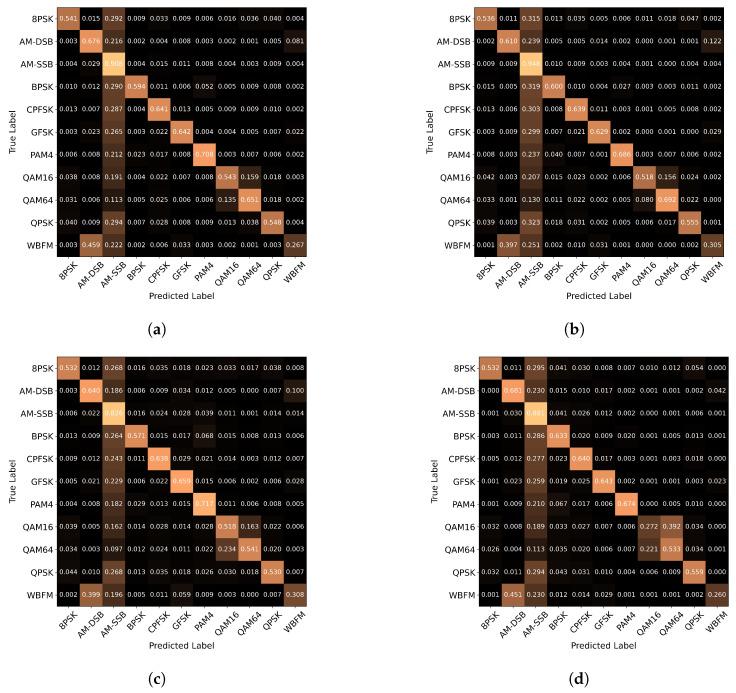

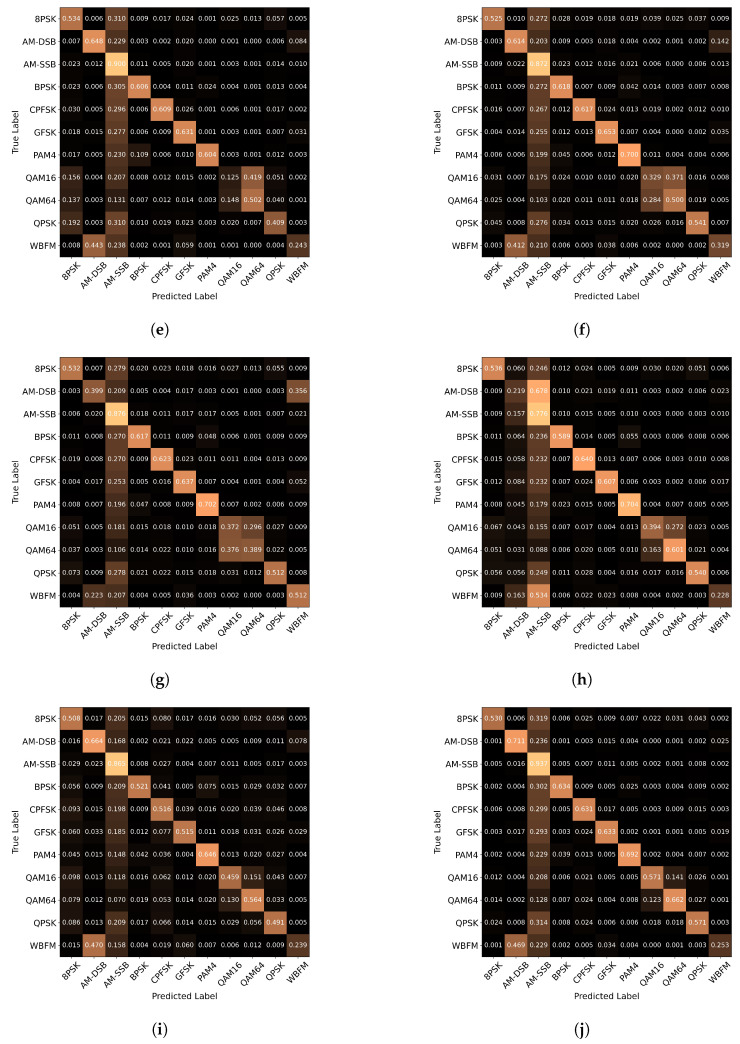
Confusion matrices of the different models on RML2016.10a. Each row of the confusion matrix corresponds to the ground truth class, while each column corresponds to the class predicted by the respective models. (**a**) AvgNet; (**b**) MCLDNN; (**c**) Resnet1d; (**d**) VGG; (**e**) CNN2d; (**f**) GRU; (**g**) LSTM; (**h**) GAF; (**i**) ConsCNN; (**j**) CTGNet.

**Figure 8:**
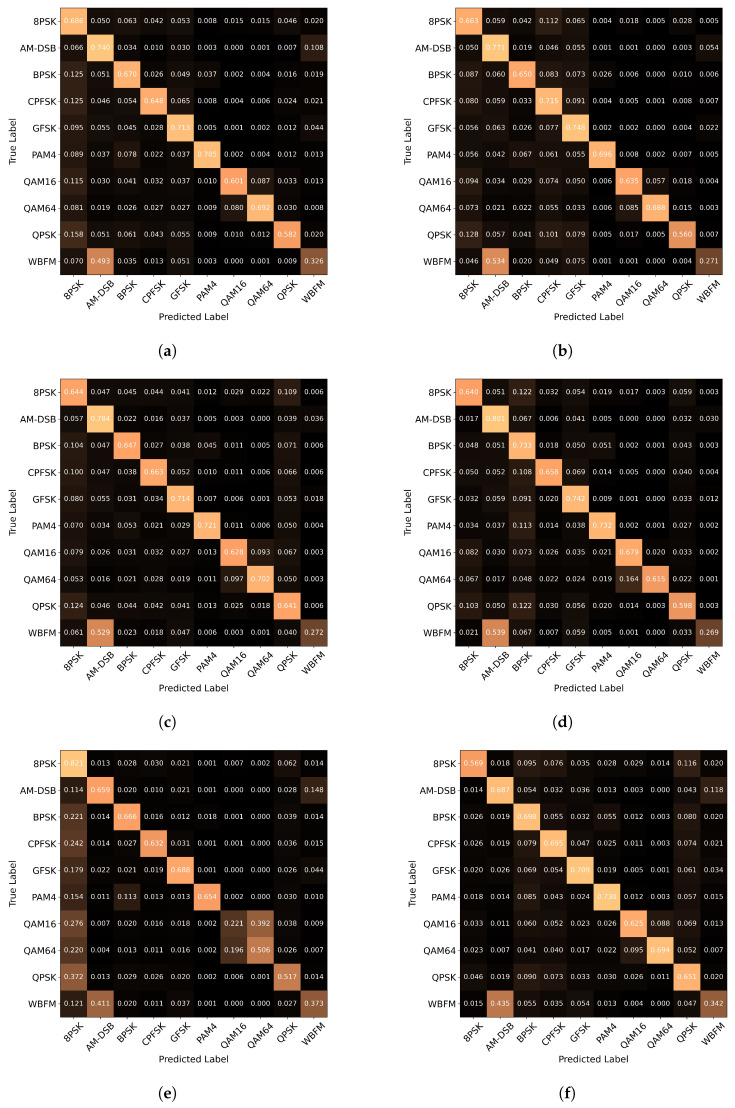

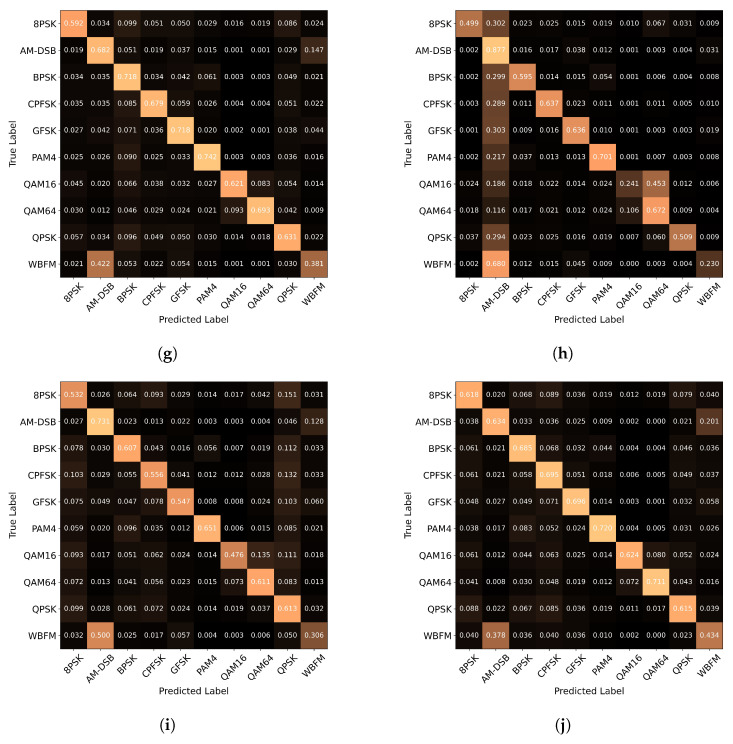
Confusion matrices of the different models on RML2016.10b. Each row of the confusion matrix corresponds to the ground truth class, while each column corresponds to the class predicted by the respective models. (**a**) AvgNet; (**b**) MCLDNN; (**c**) Resnet1d; (**d**) VGG; (**e**) CNN2d; (**f**) GRU; (**g**) LSTM; (**h**) GAF; (**i**) ConsCNN; (**j**) CTGNet.

We also show the confusion matrices obtained using different recognition methods on the two datasets, as shown in Figure 7 and Figure 8. For the RML2016.10a dataset, it can be observed that all models tended to misclassify the other modulation types as AM-SSB. Additionally, except for CTGNet, AvgNet, MCLDNN, and ConsCNN, the other methods were more prone to misclassify QAM16 and QAM64. Furthermore, except for LSTM and GAF, the other methods tended to misclassify WBFM as AM-DSB, while LSTM more frequently misclassified AM-DSB as WBFM, and GAF misclassified AM-DSB as AM-SSB. In the case of CNN2d, it tended to misclassify QAM16, QAM64, and QPSK as 8PSK. For the RML2016.10b dataset, all methods tended to misclassify WBFM as AM-DSB. CNN2d was more inclined to misclassify other modulation types as 8PSK. In comparison to the other methods, CTGNet, AvgNet, and MCLDNN showed a better ability to differentiate between QAM16 and QAM64.

#### 4.4.4. Discussion

As can be seen from the experiments in Section 4.4.1 and Section 4.4.2, the partition of subsequences of varying lengths and the amount of overlap between adjacent subsequences had an impact on the final recognition accuracy. Appropriately overlapping adjacent subsequences can bring several advantages when selecting window sizes and strides. First, overlapping adjacent subsequences implies the existence of overlapping regions, which leads to these regions receiving more attention when constructing the adjacency matrix. This facilitates increased information interaction between features within the sequence, aiding in extracting richer feature representations. Second, this can enhance contextual information. By overlapping adjacent subsequences, the model can consider the contextual information before and after the current sequence. This provides a more comprehensive contextual view, enabling the model to better understand the temporal relationships and semantic information within the sequence.

Compared with LSTM, GRU, and different CNN network models, our CTGNet transforms the signal into a graph structure and offers several advantages. By doing so, the model can capture the direct topological relationships between the sample points, enabling the model to effectively capture complex dependencies and intricate patterns. This is particularly beneficial for time series data, where long-range dependencies and non-linear relationships are prevalent. AvgNet, which constructs the graph structure using one-dimensional convolutions, is constrained by the convolutional kernel size, resulting in the oversight of long-range dependencies between sample points. In contrast, our proposed model not only captures local relationships, but also considers global contextual information. This comprehensive approach enhances the construction of topological relationships within time series data. Furthermore, our end-to-end model dynamically constructs the graph topology of the signal, enabling it to achieve optimal recognition accuracy. By leveraging the graph structure, our model could effectively integrate contextual information, capture complex dependencies, and adaptively represent the data, leading to superior performance compared to the baseline methods. In summary, our proposed CTGNet had the best performance among all the baseline methods, with a reasonable model size and training time. The model benefits from its ability to capture complex dependencies, incorporate contextual information, and dynamically construct a graph topology. These features collectively empowered our model to achieve exceptional recognition accuracy on both datasets.

At the same time, our proposed CTGNet has the potential limitation of a large computational complexity. In addition, when we partitioned the IQ signal, we used fixed lengths and steps. In the future, we will continue to study and devote ourselves to designing lightweight models to reduce computational complexity and design data preprocessing methods with variable parameters, such as a sliding window length and steps to improve model performance.

## 5. Conclusions

In this study, we introduced CTGNet, a novel approach that adaptively transforms signals into a graph structure for effective modulation classification. The proposed method comprises several key steps. First, we preprocess the raw IQ signal using a sliding window of fixed size and stride, obtaining subsequence samples. Next, we proposed a CTN to construct a graph structure based on these extracted subsequences. Finally, a graph neural network is utilized for the classification task. This end-to-end model demonstrated the capability to learn an optimal graph topology specifically tailored for signal classification. To validate the effectiveness of CTGNet, we conducted extensive experiments on the RML2016.10a and RML2016.10b datasets. Our results showcased the superiority of our proposed model in accurately classifying modulations. Additionally, we investigated the impact of different partition lengths of subsequences and the degree of overlap between adjacent subsequences on the recognition accuracy. The method proposed in this paper provides a reference value for the field of automatic modulation classification; that is, to explore different representation methods of signals and mine the potential features contained in them. In the future, different representation methods of signals, such as amplitude/phase, time–frequency representation, and graphs, can be fused to provide more abundant information, to promote the improvement of recognition performance.

## Figures and Tables

**Table 1:** The structure of CTGNet. The length of the sliding window in data preprocessing is 16, and the step size is 8.

Operation	Output Dimensions
Input	128 × 1
Data Preprocessing	15 × 16
Conv1D (size 5)	15 × 96
Position Encoding	15 × 96
TransformerEncoderLayer (num_layers 3, $n_{h}$ 8, $d_{k}$ 96, $d_{v}$ 96)	15 × 96
Multi-Head Attention + Average	adjacency matrix: 15 × 15
Concatenate subsequences of I and Q	node feature: 15 × 32
GraphSAGE + BN + Relu	15 × 96
GraphSAGE + BN + Relu	15 × 96
GraphSAGE + BN + Relu	15 × 96
GraphSAGE + BN + Relu	15 × 96
GraphSAGE + BN + Relu	15 × 96
GraphSAGE + BN + Relu	15 × 96
Concatenate the output of above 6 layers	15 × 576
DMoNPool	node feature: 15 × 576, adjacency matrix: 15 × 15
GraphSAGE + BN + Relu	15 × 96
GraphSAGE + BN + Relu	15 × 96
GraphSAGE + BN + Relu	15 × 96
GraphSAGE + BN + Relu	15 × 96
GraphSAGE + BN + Relu	15 × 96
GraphSAGE + BN + Relu	15 × 96
Concatenate the output of above 6 layers	15 × 576
DMoNPool	node feature: 8 × 576, adjacency matrix: 8 × 8
GraphSAGE + BN + Relu	15 × 96
GraphSAGE + BN + Relu	15 × 96
GraphSAGE + BN + Relu	15 × 96
GraphSAGE + BN + Relu	15 × 96
GraphSAGE + BN + Relu	15 × 96
GraphSAGE + BN + Relu	15 × 96
Concatenate the output of above 6 layers	15 × 576
Average	576
FC + Relu	288
FC + Relu	96
Concatenate feature vectors $T_{I}$ and $T_{Q}$	192
FC + Relu	96
FC + Softmax	modulation modes

**Table 2:** The parameters of the two datasets used in the experiments. The SNR of both datasets ranges from −20 to 18 dB, with RML2016.10a containing 11 modulation modes and RML2016.10b containing 10 modulation modes.

Dataset	SNR	Modulation Types
RML2016.10a	−20 dB:2 dB:18 dB	BPSK, QPSK, 8PSK, 16QAM, 64QAM, GFSK, CPFSK, PAM4, WBFM, AM-SSB, AM-DSB
RML2016.10b	−20 dB:2 dB:18 dB	BPSK, QPSK, 8PSK, 16QAM, 64QAM, GFSK, CPFSK, PAM4, WBFM, AM-DSB

**Table 3:** Recognition performance on two datasets with/without data preprocessing.

Dataset	Model	Accuracy	F1 Score	Recall	Model Size (MB)	Training Time (s)
RML2016.10a	w/processing	0.6207	0.6439	0.6207	4.62	0.0440
	wo/processing	0.6168	0.6378	0.6168	9.55	0.2446
RML2016.10b	w/processing	0.6433	0.6463	0.6433	4.62	0.0444
	wo/processing	0.6418	0.6393	0.6418	9.55	0.2436

**Table 4:** Recognition accuracy, F1 score, and recall of ten methods on the RML2016.10a dataset.

Model	Accuracy	F1 Score	Recall	Model Size (MB)	Training Time (s)
AvgNet	0.6112	0.6309	0.6112	3.63	0.0897
MCLDNN	0.6107	0.6370	0.6107	1.94	0.0219
Resnet1d	0.5891	0.6037	0.5891	0.91	0.0072
VGG	0.5732	0.5867	0.5732	47.32	0.0730
CNN2d	0.5286	0.5344	0.5286	10.91	0.0082
GRU	0.5720	0.5870	0.5720	2.52	0.0085
LSTM	0.5614	0.5773	0.5614	0.97	0.0111
GAF	0.5309	0.5571	0.5309	63.15	0.1547
ConsCNN	0.5437	0.5449	0.5437	69.22	0.3006
CTGNet	0.6207	0.6439	0.6207	4.62	0.0440

**Table 5:** Recognition accuracy, F1 score, and recall of ten methods on the RML2016.10b dataset.

Model	Accuracy	F1 Score	Recall	Model Size (MB)	Training Time (s)
AvgNet	0.6363	0.6406	0.6363	3.63	0.0858
MCLDNN	0.6394	0.6412	0.6394	1.94	0.0219
Resnet1d	0.6315	0.6298	0.6315	0.91	0.0078
VGG	0.6365	0.6350	0.6365	47.32	0.0709
CNN2d	0.5564	0.5638	0.5564	10.91	0.0080
GRU	0.6307	0.6298	0.6307	2.52	0.0088
LSTM	0.6329	0.6332	0.6329	0.94	0.0114
GAF	0.5598	0.5766	0.5598	63.15	0.1685
ConsCNN	0.5629	0.5641	0.5629	69.22	0.2998
CTGNet	0.6433	0.6463	0.6433	4.62	0.0444

## Data Availability

Not applicable.
